# Can Inflammatory and Nutritional Serum Markers Predict Chemotherapy Outcomes and Survival in Advanced Stage Nonsmall Cell Lung Cancer Patients?

**DOI:** 10.1155/2019/1648072

**Published:** 2019-02-28

**Authors:** Ruksan Cehreli, Tugba Yavuzsen, Halil Ates, Tulay Akman, Hulya Ellidokuz, Ilhan Oztop

**Affiliations:** ^1^Institute of Oncology, Department of Preventive Oncology, Dokuz Eylul University, Inciralti, Izmir 35340, Turkey; ^2^Division of Oncology, Dokuz Eylul University School of Medicine, Inciralti, Izmir 35340, Turkey; ^3^Hematology Laboratory, Dokuz Eylul University Institute of Oncology, Izmir, Turkey; ^4^Division of Medical Oncology, Kent Hospital Izmir, Turkey

## Abstract

*Purpose. *To determine the values of prognostic nutritional and inflammatory markers in chemotherapy outcomes and survival in the patients with advanced nonsmall cell lung cancer (NSCLC) and also in the secondary malnutrition and cachexia.* Methods. *Twenty-five patients with diagnosis of aNSCLC were registered for the prospective study. Malnutrition was determined by the Subjective Global Assessment (SGA) and performance status by criteria of the Eastern Cooperative Oncology Group (ECOG). Before treatment, serum levels of albumin, prealbumin, vitamin D, zinc (Zn), C-reactive protein (CRP), IL-6, IL-1 *β*, TNF-*α*, lipoprotein lipase (LPL), and the Glasgow Prognostic Score (GPS) were recorded. Patients were followed prospectively for treatment outcomes and survival.* Results. *Due to the deaths of 18 patients during the 4-month follow-up period, no adequate measurements of inflammatory and nutritional markers could be performed. However, seven patients completed the treatment period and evaluations of these markers could be performed during the three periods. Eighty-four percent of patients were male with a mean age of 63.3 ± 8.7 years. Evaluation of the malnutrition by SGA showed that 5 (20%) patients were well nourished (A), 12(48%) were moderately malnourished (B), and 8(32%) were severely malnourished (C). Low levels of serum albumin (<3.5g/dl), prealbumin (<20 mg/ml), 25-hydroxycholecalciferol (<30 ng/ml), and Zn (<70mg/ml) were detected in 15(60%), 17(68%), 24 (96%), and 22 (88%) patients, respectively. Elevated levels of CRP (≥10 mg/L), IL6 (≥18pg/ml), TNF-*α* (≥24pg/ml), IL-1*β* (≥10pg/ml), and LPL (<12pg/ml) were found in 24 (96%), 11(44%), 9(36), 13(52%), and 11(44%) patients, respectively. Moderate and severe malnutrition, acute phase response, and reduced survival were determined in patients with NCSLC. In 7 patients that completed the treatment period, there was an association between elevated serum levels of IL-6, IL-1*β*, TNF-*α*, CRP, and LPL and also the reduced serum levels of albumin, prealbumin, Zn, vitamin D, and GPS, respectively. Similarly, Friedman analysis indicated that prealbumin significantly increased (p=0.007) in the follow-up period. But the serum levels of CRP (mean 37.3±22.3; Wilcoxon test P=0.368) in the seven patients were lower than those of the 18 patients that expired (mean 75.82±56.2).* Conclusion*. Malnutrition and cachexia negatively influence oncological outcomes in patients with NSCLC. These nutritional/inflammatory markers may be useful for selection of high risk and reduced survival in patients with aNSCLC undergoing adjuvant chemotherapy.

## 1. Introduction

Malnutrition is a complex and major problem encountered in patients with each stage of the cancer. Adverse effects due to cancer or treatment can lead to or exacerbate the process of malnutrition. Anorexia Cachexia Syndrome (ACS) is a clinical and metabolic disorder, accompanied by an involuntary weight loss and is associated with morbidity and mortality in cancer patients [1]. The overall prevalence of ACS ranges from 40 to 80% in newly diagnosed and in advanced stages of cancer respectively [2].

Furthermore malnutrition is commonly caused by multiple deficiencies of nutrients, several vitamins and minerals (i.e., folic acid, B6, B12, A,C,E,D, riboflavin, iron, and zinc). Previous reports suggest that inadequate immune response is a consequence of nutrient deficiency [3–5]. In addition to these, zinc is an essential element for normal functioning of cells, such as neutrophils, natural killer cells, CD4, and CD8. Zinc decreases oxidative stress markers (i.e., CRP) and generation of inflammatory cytokines. It is also known as antioxidant by increasing the body immunity [4–6]. Also, zinc is transported to tissues by the blood proteins albumin and alpha-2 macroglobulin [7]. During recent years, vitamin D deficiency has been commonly described in patients with cancer. Vitamin D is the precursor of the potent steroid hormone calcitriol that regulates numerous cellular pathways and plays a role in determining cancer risk and prognosis also shows beneficial anti-inflammatory process in several cancers. It is also involved in some key mechanisms including inhibition of proinflammatory cytokine production and NF *κ*B signaling [5, 6, 8]. Additionally, studies have shown that chemotherapy causes decrease in T cell numbers by reducing serum zinc and vitamin D levels and resulting in suppressing cell-mediated and adopted immunity [6–8]. In a systematic review, including 10 studies on lung cancer, the majority of studies identified that higher albumin is associated with increased survival [9, 10].

Inflammation is one of the multistep mechanisms in induction of cancer cachexia. In cancer patients, increased levels of the blood cytokine (i.e., IL-1*β*, IL-6, and TNF*α*) have been recognized; however, these cytokines contribute most likely to a small portion of the abnormal metabolism [1, 6, 7, 9].

In recent years, much evidence has supported that systemic inflammation is related to shorter survival among patients with many cancers through promotion of cancer cell proliferation and survival and tumor metastasis.

The Glasgow Prognostic Score (GPS) is CRP and albumin, which represent not only inflammatory status but also nutritional status. Levels of CRP and albumin have been considered as possible prognostic factors of cachexia development for patient with cancers [6, 9, 10].

LPL is processed ubiquitously in multiple tissues and its expression has been extensively studied. Furthermore, the lowest LPL activity was inversely proportional to the greatest percent of weight loss in patients with lung cancer [11]. Another report revealed that high LPL activity in aNSCLC tissue strongly predicted reduced patient survival [12].

Numerous studies have confirmed the relationship between inflammation and cancer cachexia and prognosis. Also incidence of cancer cachexia has been estimated to be 60% in patients with lung cancer. Depending on the above-mentioned findings, the tools and principles to be used in screening of cancer cachexia are needed. Progressive weight loss is inevitable in patients with end-stage cancer, and early nutritional therapy is important for effective treatment, inflammatory response, and tumor control. By providing patients with medical nutrition therapy, tolerance to cancer treatments and support to immune system can contribute to a better quality of life [5, 9, 10].

The aim of this prospectively designed study is to investigate the association between nutritional and serum inflammatory biomarkers and the effectiveness of nutritional support on cancer patients with malnutrition undergoing chemotherapy and also to determine the effect of these biomarkers on clinical outcomes in 30 patients with aNSCLC. Moreover, no study has reported the relation of Glasgow Prognostic Score (GPS) to serum levels of zinc and vitamin D as an anti-inflammatory and antioxidative agent in patients with cancer. Additionally, with a different approach, we considered that evaluating GPS with serum levels of zinc and vitamin D together with all nutritional and inflammatory biomarkers (such as proinflammatory cytokines, LPL, CRP, albumin, prealbumin, SGA) utilized in the previous studies reported in the literature might be useful and meaningful for estimating prognostic, nutritional, and inflammatory markers in patient with aNSCLC.

## 2. Materials and Methods

### 2.1. Study Design

Approval for the study was obtained from the local ethics committee (ethical approval number: 176-İOÇ) of the Dokuz Eylul University Faculty of Medicine and written consent forms were acquired for all cases. Between November 2012 and June 2013, newly diagnosed stage 3B and 4 NSCLC patients were prospectively enrolled for the study. A target number of 30 patients within 2 months was considered. The completion time of recruitment with a total follow-up period after the study was determined to be 10-12 months. However, 5 patients refused to participate in the study.

#### 2.1.1. Inclusion Criteria

1. Eligible patients were required to have chemotherapy and/or radiotherapy naïve and age ≥18 years.
2. Performance status ≤3 by ECOG.
3. Life expectancy is at least 4 months.
4. BMI (≤20) was evaluated.

#### 2.1.2. Exclusion Criteria

1. Patients with preexisting thrombophlebitis or a history of deep vein thrombosis
2. Impaired food intake and long-term drug use
3. Patients who were unwell to be engaged in this program

Newly diagnosed stage 3B and 4 NSCLC 25 patients underwent assessment for nutritional status by SGA before chemotherapy. Following the evaluation of the patients with a BMI ≤ 20, the nutritional support composed of liquid-based nutrient solution supplemented with appropriate omega-3 fatty acids (1.5g/d), high protein was also provided by addition of megestrol acetate (160-480 mg), and nutritional counseling was initiated.

### 2.2. Outcome Measures

Routine laboratory evaluations from patient's blood samples included albumin, prealbumin, CRP, zinc, and vitamin D3 at baseline; 2- and 4-month time points following chemotherapy were scheduled.

25-Hydroxycholecalciferol was analyzed by chemiluminescence (Siemens ADVIA Centaur XP) and CRP and Zn by an autoanalyzer device.

The GPS, based on CRP and albumin levels, was 2 when levels of both CRP >10 mg/L and albumin <3.5g/dl were present and 1 when only one abnormality is observed, and 0 when both levels were normal [13]. A score of 1 or more was considered abnormal.

### 2.3. Serum Cytokine and LPL Analysis by ELISA

Blood samples were collected by venipuncture at diagnosis and at the 4-month follow-up. Blood samples were centrifuged at 1,000 x g for 15 minutes followed by separation of plasma and serum into equal aliquots. Aliquots were stored at -80°C indefinitely until time of analysis. Plasma or serum IL-1*β*, IL-6, TNF-*α*, and LPL were determined with quantitative ELISA kits (Boster Immunoleader and Cusabio Biotech Co., Ltd) using a BioTek.

Synergy HT microplate reader (Conquer Scientific Lab Equipment, CA, USA) was used. The calculations were performed based on the manufacturer's recommendation. The results were shown as pg/ml for IL-1*β*, IL-6, and TNF-*α* and ng/ml for LPL.

### 2.4. Statistical Analysis

Variables specified with measurement were summarized as arithmetic means with standard deviation (SD) and medians with minimum maximum values analyzed by nonparametric tests. Wilcoxon test was used in the analysis of two replicates and more than two replicates of the Friedman test were used. A* p* value of less than 0.05 was considered significantly different. Statistical analysis was done by using SPSS v22.0 for Windows.

## 3. Results

The patient demographics and baseline values are shown in Table 1. Twenty-five patients were enrolled in the study. Eighteen patients died during the study follow-up period; therefore, seven patients were evaluated. Of the patients, 84% were male (mean age 63.3±8.7 and BMI of 20.8±2.6 kg/m^2^). At the time of SGA, 20% of the patients were well nourished (A), whereas 48% of participants were moderately malnourished (B) and 32% were severely malnourished (C). Regarding nutritional and inflammatory markers, the mean serum albumin level was 3.6±0.3 g/dl (range: 3.5-5.2 g/dl). Eleven patients (44%) had pretreatment level less than or equal to 3.5 g/dl. The mean serum prealbumin level was 19.0±3.8 (range: 19-38 mg/dl) and seventeen patients (68%) had pretreatment prealbumin level of less than or equal to 20 mg/dl.

We also examined serum levels of IL-1*β*, IL-6, TNF-*α*, and LPL to determine the association with inflammation and clinical outcomes of patients with NSCLC. The CRP median was 54.4 mg/L and 24 patients (96%) had a CRP level ≥10 mg/L [1]. Serum contents of TNF-*α*, IL-6, and IL-1*β* were 36.15±36.6, 26.0±29.4, and 15.13±24.2 pg/ml, respectively. Due to lack of a consensus for optimal cut-off level, we stratified patients depending on median cytokine level for analysis in this study (see Table 1) [8]. A CRP level greater than 10 mg/L correlated with advanced tumor stage and reduced albumin and survival. Also patients with IL-6 ≥18 pg/ml or TNF-*α* ≥24 pg/ml had a reduced albumin and survival (Table 1).

Also, serum 25-hydroxycholecalciferol level was 16.7±6.6 mg/dl (range: 30-100 ng/ml) as a nutritional marker. Vitamin D level was <30 ng/ml in 25 patients and Zn was 54.7±14.8 mg/dl (range: 70-115 mg/ml) in 22 patients (88%) that had pretreatment ≤70 mg/ml (Table 1). There were a significantly higher serum 25-hydroxycholecalciferol and zinc levels in seven patients during the three follow-up periods 18.1±4.9, 18.7±6.8, and 17.5±5.3 (*p*=0.651) and 56.8±14.2, 70.2±10.9, and 63.6±12.9 (*p*=0.102), respectively, in this study (Table 2).

Serum LPL level was 21.37±24.2 ng/ml (median: 11.55 ng/ml) and 11 patients (44%) had pretreatment serum level ≥12 ng/ml (Table 1). However, serum LPL levels in the seven patients were significantly lower during in the follow-up periods 20.3±12.5 and 15.4±8.2 (*p*=0.018) (Table 2).

Twenty patients (80%) had GPS of 2 (Table 1). On the other hand, seven patients had a GPS of 1 (only CRP >10 mg/L) (Table 2). We defined this score value as a predictor for survival. Furthermore, increased CRP levels (>10 mg/L) were inversely proportional to albumin levels. Of the 7 subjects that were able to complete the follow-up period, 71.4% (5) were male and 28.6% (2) were female. Presently, 5 patients deceased and only 2 remained alive (Table 2). At the same time, in the seven living patients, the mean level of the CRP levels in the 3 periods were slightly higher (37.3±22.3, 15.4±13.6, 35.8±3.9, respectively (*p*=0.368).

However, the values of proinflammatory cytokines and LPL showed a significant decrease within the 4-month follow-up period. IL-6 had a mean of 16.7±12.0 and 4.7±5.0 (*p*=1.0), IL-1*β* had a mean of 14.6±15.5 and 16.7±11.9 (*p*=0.018), and TNF-*α* had a mean of 53.5±38.1 and 20.9±11.2 (*p*=0.028). Also, LPL had a mean of 20.3±12.5 and 15.4±8.2 (*p*=0.018).

In the present study, Wilcoxon/Friedman tests indicate a positive association between inflammation and malnutrition, and the serum contents of the inflammatory cytokines in patients with NSCLC. However, these tests were only administered in seven patients who were capable of continuation of treatment.

There were significantly higher albumin and prealbumin levels in the seven patients during the follow-up periods 4.07±0.2, 4.02±0.2, 4.04±0.2 (*p*=0.857) and 19.0±3.8, 28.4±7.8, 21.1±5.9 (*p*=0.007), respectively (Table 2).

Particularly, the prealbumin and albumin may be useful as nutritional biomarkers for evaluation of survival.

## 4. Discussion

The presented study evaluated inflammatory markers in the prediction of chemotherapy outcomes in patients with advanced stage NCSLC that were followed for 4 months. The findings of our study demonstrate that occurrence of malnutrition and cachexia is related to low SGA and BMI [14].

BMI and SGA have been used to evaluate nutritional status. In our patients, the mean BMI was 20.8 kg/m^2^; if this value was considered alone, no malnutrition was reflected in this index according to the WHO definition. However, 80% of our patients were indeed malnourished as measured by other parameters such as SGA. These results suggest that BMI is not a sufficient parameter because body weight is heavily influenced by fluid retention and edema [15, 16].

SGA is the reference method used to assess nutritional status in cancer. This scale determines severe or moderate malnourishment or well-nourishment [10, 14, 16].

Malnutrition status measured by SGA demonstrated a significant connection with physical functioning and symptoms including loss of appetite, dyspnea, fatigue, and diarrhea. For this reason, patients' nutrition counseling and nutritional supplementation were given. However, it was observed that the patients did not get enough supplementation.

Serum albumin has been used to determine nutritional status. Reduced albumin levels (< 3.5 g/dL) often reflect malnutrition and may predict patient survival. However, reduced albumin was prognostic in GPS in our study [13, 17]. Recovery of albumin level is important after chemotherapy because nutrition deficiency is higher in advanced stage of cancer patients with malnutrition. In a systematic review, including 10 studies on lung cancer, the majority of studies identified that higher albumin is associated with increased survival [9, 10, 18], which is consistent with our results. However, albumin levels were a significant prognostic variable.

A probable explanation of this effect may be suppression in albumin synthesis by malnutrition and inflammation in an advanced stage of lung cancer [19].

Prealbumin, also known as transthyretin, has a half-life in plasma of ~2 days. It is therefore more sensitive to changes in protein-energy status than albumin and it is used as a nutritional biomarker closely reflecting overall nutritional status [10, 18]. Similarly with albumin, our study identified that higher prealbumin levels are associated with increased survival.

The Nutritional Risk Index (NRI) includes serum albumin and normal and current weight. These tools are not specific for cancer patients [14, 19, 20]. For this reason, NRI is not preferred and future validation of specific tests for cancer patients is needed.

CRP has been reported to be associated with reduced serum albumin, resulting in progressive weight loss, poor performance, and higher mortality in cancer patients with systemic inflammation. Therefore, both CRP and serum albumin have been reported to be associated with elevated CRP, IL-6, TNF-*α*, IL-1*β*, and zinc deficiency [10, 19, 21–23].

Recently, Hamilton and colleagues showed that elevated CRP level was associated with inflammatory status as a prognostic factor for advanced stage NCSLC patients [24]. IL-6 level seems to be directly proportional to late stage of patients with cancer. Previous reports have shown an association between circulating IL-6 level and the survival. Future studies are required for prognostic value of IL-6 [22, 24–27].

LPL activity has been shown in NSCLC and predicts patient survival [11, 12, 28]. However, until present serum LPL levels have not been examined in patients with aNCSLC. In our study determination of LPL activity could not be performed in all patients because of the death of a large proportion of patients and statistical significance could not be reached. Also, we think that further large-scale prospective studies are required in order to establish serum LPL marker.

Zinc deficiency can cause an increase in oxidative stress and proinflammatory cytokines in patients with malnutrition and cachexia. Previous reports have shown that free intracellular zinc resulted in increased sensitivity of dendritic cells to CD4 T-cells activation [5, 29–33], which is consistent with our studies.

In our study, pretreatment Zn was deficient (54.7±14.8mg/dL) in 22 patients. There were significantly higher levels of serum Zn in seven patients during the follow-up period in this study as compatible with the levels of albumin and CRP.

Vitamin D deficiency has been shown as a negative predicting factor in the elderly studies [34]. Vitamin D deficiency has been shown to be associated with reduced rates of progression-free survival and overall survival in Hodgkin lymphoma patients [35]. Similar results reported that 25(OH) D deficiency is an independent prognostic factor for poor survival in patients with advanced stage of NSCLC treated with chemotherapy [36]. Our study revealed more evidence for vitamin D as a prognostic marker in NSCLC.

In the presented study we showed that elevated levels of CRP, IL-6, IL-1*β*, and TNF-*α* and decreased levels of albumin, prealbumin, Zn, vitamin D, BMI, and high GPS play a role in predicting chemotherapy outcomes including morbidity and mortality. Lower levels of zinc and 25-cholecalciferol levels are associated with high levels of CRP and proinflammatory cytokines, IL6, IL-1*β*, TNF-*α* because increased serum levels of proinflammatory cytokines and CRP for 6 weeks may cause decreases in serum zinc levels [7].

An essential inflammation mediator downstream targeted medical nutrition containing high-dose of omega-3 fatty acids, vitamin D, Zn, and high-quality protein is well tolerated with a good safety profile and has positive effects on immune system and survival. However, more clinical studies are needed [37–39].

Given all those findings, patients receiving cancer treatment and bearing a risk of malnutrition and cachexia must be evaluated in terms of micronutrients. Research has shown that early medical nutrition therapy may help improving the negative nitrogen balance, promote immune function, and reduce the incidence of complications [10, 37, 39, 40].

As a result, analysis of 25 patients with advanced stage of NSCLC according to GPS values in our study revealed that patients with a high GPS showed higher treatment toxicity and lower survival.

In this study, especially in relation to high GPS values and values of albumin and CRP, finding of reduced levels of Zn and 25-cholecalciferol was meaningful. We considered that measurements of these low-priced and practical parameters should be preferable in cancer patients. We were unable to use these parameters in high number of patients because our study could be performed on reduced number of patients; however, we consider that trial of these parameters in more models of studies would be beneficial.

The present study also demonstrated that CRP, albumin, GPS, and IL6 as inflammatory markers and albumin, prealbumin, Zn, 25-cholecalciferol levels as nutritional markers may be viable serum biomarkers. With further validation, these findings can be incorporated into clinical practice for counseling patient's nutrition and decision-making for treatment. More importantly, it can lead to further investigations into the nature of these biomarkers and others regarding disease biology and host response in lung cancer [39, 40].

Using these biomarkers, we may be able to better stratify patients into risk categories and subsequently adjust decision-making for treatment. Our study adds to the growing literature investigating biomarkers of malnutrition and cachexia in patients with NCSLC.

TNM staging system is used in clinical practice to determine treatment for patients with lung cancer [41]. However, this staging system sometimes fails to predict patient prognosis and may not offer appropriate clinical guidance for practice. Indeed, patients at the same TNM stage may have other conditions that influence treatment outcomes. Thus, there is a need to identify more sensitive and convenient pretreatment prognostic factors to enable more personalized treatment [41].

There are some limitations in our study. Firstly, it has a small sample size. Secondly, although in designing the prospective study most of the patients could not complete nutritional treatment due to refractory stages of cachexia, there was inability of completing treatment periods in 19 patients. In this study, it is important to screen the patients at the locally advanced and stage 3 of disease in terms of nutritional parameters and to support their early feeding. It has been thought that the patients will have less toxicity and better tumor response by evaluation of early nutritional status.

## 5. Conclusion

In conclusion, the results of our study indicate that this cannot be done only by measurement of body weight changes but, preferably, by measurements of prognostic biomarkers. Malnutrition and sarcopenia in advanced cancer patients should be evaluated before chemotherapy.

Chronic systemic inflammation and malnutrition in these patients will greatly affect the immune system. For this reason, complications and early mortality will be seen in patients undergoing chemotherapy when tumor response and over-survival are decreased. Several nutritional and inflammatory serum markers (serum, Zn, vitamin D, albumin, CRP,IL-6 level) have been investigated as predictors of systemic inflammatory status and prognosis of patients with NSCLC, but serum, Zn, vit D, albumin, and CRP are available for use in daily practice. There is a great need for practical clinical biomarkers to be able to perform this sensitive evaluation before giving medical treatment.

We think that further large-scale prospective studies are required in order to establish these biomarkers as a guide for selection of patients for treatment.

Based on the results of this study, a possible and better therapeutic strategy can be derived and implemented in patient care by the supplementation of immune-nutritional agents in patients having high GPS score, particularly with low levels of zinc and vitamin D. Also the medical nutrition can be considered in order to improve cell-mediated and adopted immunity during prechemotherapy period.

## Figures and Tables

**Table 1:** Baseline and clinical characteristics of the 25 patients with aNSCLC.

*Patients characteristics*		*Total* (n = 25)	
Age	(years, mean ± SD)	63.3 ± 8.7	
Gender	(female / male)	21 / 4	
Nutritional status (SGA)	n (%)		
	A - well-nourished	5 (20)	
	B - moderately malnourished	12 (48)	
	C - severely malnourished	8 (32)	
Weight	(kg, mean ± SD)	61.4 ± 8.6	
BMI	(kg/m^2^, mean ± SD)	20.8 ± 2.6	
Weight loss	in the last 3 months (yes / no)	19 / 6	
Albumin	mean ± SD (g/dl)	3.5 ± 0.4	
	max / min	4.4 / 2.7	
	≤ 3.5 g/dl, n (%)	15 (60)	

Prealbumin	mean ± SD (mg/dl)	19.0 ±3.8	
	max / min	25 /14	
	≤ 20 mg/dl, n (%)	17 (68)	

CRP	mean ± SD, median (mg/L)	75.8 ±56.2	54.4
	max / min	194 /5.8	
	≥10 mg/L, n (%)	24 (96)	

IL-6	mean ± SD, median (pg/ml)	26.0 ± 29.4	17.5
	max / min	148.4 / 5.4	
	≥ 18 pg/ml, n (%)	11 (44)	

IL-1*β*	mean ± SD, median (pg/ml)	15.1 ± 24.3	9.7
	max / min	100 / 0.61	
	≥ 10 pg/ml, n (%)	9 (36)	

TNF-*α*	mean ± SD, median (pg/ml)	36.2 ± 36.6	24.6
	max / min	120 / 1.5	
	≥ 24 pg/ml, n (%)	13 (52)	

LPL	mean ± SD, median (ng/ml)	21.37 ± 24.2	11.6
	max / min	105 / 3.8	
	> 12 ng/ml, n (%)	11 (44)	

Zinc	mean ± SD, median (mg/ml)	54.7 ± 14.8	53.2
	max / min	95.8 / 36.2	
	≤ 70 mg/ml, n (%)	22 (88)	

25-Hydroxycholecalciferol	mean ± SD, median (ng/ml)	16.7 ± 6.6	15.2
	max / min	34.8 / 7.3	
	≤ 30 ng/ml, n (%)	24 (96)	
GSP n (%)	Both normal (CRP ≤10 mg/L, Albumin ≥3.5 g/dl)		1 (4)
	One abnormal (CRP ≤10 mg/L, Albumin <3.5 g/dl)		4 (16)
	Both abnormal (CRP >10 mg/L, Albumin <3.5 g/dl)		20 (80)

*n*: number, *SGA*: subjective global assessment, *max/min* = maximum/minimum, *BMI*: body mass index, *CRP*: C-reactive protein, *IL6*: interleukin 6, *IL1-β*: interleukin 1*β*, *TNF-α*: tumor necrosis factor alpha, *LPL*: lipoprotein lipase, *GPS*: Glasgow prognostic score.

**Table 2:** Association between nutritional and inflammation parameters of the seven patients with advanced NSCLC.

Parameters	Prechemotherapy	2-month	4-month	*P*
*Albumin*				
mean ± SD	4.07 ± 0.2	4.02 ± 0.2	4.04 ± 0.2	*p *=0.857*∗*
median (min/max)	4.0 (3.8/4.4)	4.0 (3.7/4.5)	3.9 (3.7/4.5)	

*Prealbumin*				
mean ± SD	19.0 ± 3.8	28.4 ± 7.8	21.1 ± 5.9	*p *= 0.007*∗*
median (min/max)	19.0 (14/25)	27.0 (18/40)	23 (11/29)	

*CRP*				
mean ± SD	37.3 ± 22.3	15.4 ± 13.6	35.8 ± 3.9	*p *= 0.368*∗*
median (min/max)	31 (18.9/79.9)	12.3 (1.8/36.1)	29.5 (3.8/83.5)	

*25-Cholecalciferol*				
mean ± SD	18.1 ± 4.9	18.7 ± 6.8	17.5 ± 5.3	*p *= 0.651*∗*
median (min/max)	17.6 (10.3/24.3)	19.6 (10/31.5)	17.3 (8.7/24.6)	

*Zinc*				
mean ± SD	56.8 ± 14.2	70.2 ± 10.9	63.6 ± 12.9	*p *= 0.102*∗*
median (min/max)	64.7 (39.9/72.3)	68.3 (59.6/89.7)	59.8 (47/80.3)	

*IL-6*				
mean ± SD	16.7 ± 12.0		4.7 ± 5.0	*p *= 1000*∗∗*
median (min/max)	13.3 (5.4/40.1)		16.4 (5.6/38.7)	

*IL-1β*				
mean ± SD	14.6 ± 15.5		16.7 ± 11.9	*p *= 0.018*∗∗*
median (min/max)	14.2 (0.7/46.7		0.7 (0.6/11.8)	

*TNF-α*				
mean± SD	53.5 ± 38.1		20.9 ± 11.2	*p *= 0.028*∗∗*
median (min/max)	46.7 (14.1/120)		17.1 (5.6/38.4)	

*LPL*				
mean ± SD	20.3 ± 12.5		15.4 ± 8.2	*p *= 0.018*∗∗*
median (min/max)	16.7 (7.5/44.5)		12.3 (6.3/30)	

*GPS* n (%)	One abnormal (CRP >10 mg/L) score 1		7 (100 %)	

*n*: number, *CRP*: C-reactive protein, *IL6*: interleukin 6, *IL1-β*: interleukin 1*β*, *TNF-α*: tumor necrosis factor alpha, *LPL*: lipoprotein lipase, *∗p* values were based on Wilcoxon test, *∗∗* and Friedman test.

## Data Availability

The data used to support the findings of this study are available from the corresponding author upon request.
